# Pediatric massage in conjunction with other traditional Chinese medicine therapies for tic disorder in children: systematic review and network meta-analysis

**DOI:** 10.3389/fped.2025.1609934

**Published:** 2025-08-26

**Authors:** Jiayue Liu, Hanyu Zhang, Tianyuan Yu, Jinping Chen, Yingqi Zhang, Jiawei Sun, Yue Xu, Rentuya Na, Jiawang Yan, Hongzheng Zhang, Mengqian Lu

**Affiliations:** School of Acupuncture-Moxibustion and Tuina, Beijing University of Chinese Medicine, Beijing, China

**Keywords:** tic disorder, pediatric massage, traditional Chinese medicine, systematic review, network meta-analysis

## Abstract

**Background:**

Pediatric massage (PM) belongs to traditional Chinese medicine treatment (TCM) and is an alternative therapy for tic disorders (TD). This systematic review and network meta-analysis (NMA) was undertaken to evaluate the efficacy and safety of PM, the effectiveness of PM in conjunction with other TCM therapies for TD and to provide an evidence-based foundation for the clinical selection of the TD treatment regimens relating to traditional Chinese medicine.

**Methods:**

Eight databases were systematically searched from their inception to June, 2024 for randomized controlled trials (RCTs) of PM and its combination with other TCM therapies for TD. Two researchers screened the studies and extracted the data according to our inclusion criteria, and the bias assessment tool from the Cochrane Handbook was used to evaluate the quality of the included studies. Stata 17.0 software was used to perform NMA, and the efficacy and safety of PM and its combination with other TCM therapies were compared and ranked.

**Results:**

A total of 24 RCTs, covering 1,657 children with TD, were included in this NMA. There were 9 intervention measures, including PM, Western medicine (WM), PM + Chinese herbal medicine (CHM), PM + manual acupuncture (MA), PM + auricular acupuncture (AA), PM + Qigong therapy (QT), PM + cupping therapy (CT), PM + scraping therapy (ST), and PM + moxibustion (Mox). PM + CHM may be most effective at improving the total effective rate and decreasing YGTSS motor tics score and YGTSS vocal tics score; PM + MA may have the best performance in reducing the YGTSS total score; and simple PM may be the best at reducing the adverse reaction rate. The GRADE assessment showed that the overall response rate and the total YGTSS score were rated as low or very low quality due to the limitations of the original study, and other measures were rated as moderate quality. Therefore, these results need to be interpreted with caution and validated further.

**Conclusion:**

PM and its combination with other TCM therapies are effective and safe interventions for TD in children. More specifically, PM + CHM may be the best choice for improving clinical effectiveness and reducing YGTSS motor tics score and YGTSS vocal tics score, while PM + MA may be the most effective for decreasing YGTSS total score. However, due to the limitations of this study, these conclusions still need to be validated using additional high-quality RCTs.

**Systematic Review Registration:**

https://www.crd.york.ac.uk/PROSPERO/view/CRD42024553846, PROSPERO CRD42024553846.

## 1 Introduction

Tic disorder (TD) is a chronic neurological disorder that originates in childhood or adolescence, with an incidence rate as high as 0.9% in children (1). The core clinical symptoms of TD in children are repeated motor or vocal tics (2). Eighty-eight percent of TD patients have at least one other psychiatric symptom, such as attention deficit hyperactivity disorder, obsessive-compulsive disorder, or depression. These symptoms can seriously affect their quality of life and social function (3). The etiology of TD is unclear and may be related to the imbalance of neurotransmitters in the cortical-striatal-thalamo-cortical circuit (CSTC) (4). Commonly used conventional Western medicine (WM) therapy mainly includes dopamine receptor antagonists and noradrenergic agents (5). Although these drugs have been shown to be effective in the treatment of TD in children, there may be adverse effects such as drowsiness, fatigue, and extrapyramidal syndrome (EPS) that may reduce patient compliance and increase the risk of recurrence (6). Psychological therapies such as comprehensive behavioral intervention for tics (CBIT) are also effective approaches for managing TD. However, the availability of these treatments is often limited due to the insufficient number of trained practitioners (7). Therefore, safer, more effective, and more accessible therapies for TD in children are necessary.

Complementary and alternative medicine (CAM) for TD has received increasing attention (8). Pediatric massage (PM) is a recognized CAM therapy that is widely used in the treatment of TD in children and is guided by the traditional Chinese medicine (TCM) theory and modern medicine principles, using specific methods to stimulate acupoints and meridians to treat diseases (9). PM has the characteristics of being drug-free, simple to perform, highly safe, and easily accepted by children (10). PM has also been included in the diagnosis and treatment guidelines for TD (11). Furthermore in TCM, PM is considered to be the preferred external treatment for preschool children with TD (12).

In recent years, most randomized controlled trials (RCTs) evaluating PM for TD in children have involved the combination of PM and other TCM therapies, such as Chinese herbal medicine (CHM), manual acupuncture (MA), auricular acupuncture (AA), Qigong therapy (QT), cupping therapy (CT), scraping therapy (ST), and moxibustion (Mox). Previous studies have emphasized the efficacy of PM for TD in children but have only looked at the comparison between PM and WM or CHM (13). The optimal combination of TCM therapies to complement PM thus remains unknown. To some extent, this creates difficulties in selecting TD treatment regimens.

Network meta-analysis (NMA) can combine direct and indirect comparisons to evaluate and rank the efficacy and safety of multiple interventions, which has unique advantages over traditional pairwise meta-analysis (14). Therefore, NMA is particularly valuable when multiple PM combination therapies exist and direct comparative data are limited. By combining direct and indirect evidence through the NMA method, this study addresses the research gap that cannot be filled by traditional pairwise meta-analyses, in order to provide a more comprehensive evidence-based basis for selecting the best TD treatment strategy.

## 2 Methods

Our NMA adhered to the Preferred Reporting Items for Systematic Review and Meta-analysis (PRISMA) statement (15). The protocol for our NMA was registered with the International Prospective Register of Systematic Reviews (PROSPERO), number CRD42024553846 (https://www.crd.york.ac.uk/PROSPERO/view/CRD42024553846).

### 2.1 Eligibility criteria

#### 2.1.1 Types of studies

Randomized controlled trials (RCTs) investigating PM or its combination with other TCM therapies for TD in children were considered.

#### 2.1.2 Types of participants

Children (younger than 18 years) with TD diagnosed by any validated criteria (e.g., International Classification of Diseases, Diagnostic and Statistical Manual of Mental Disorders, or Chinese Classification and Diagnostic Criteria of Mental Disorders) were included.

#### 2.1.3 Types of interventions

The experimental group was administered PM, or PM combined with another TCM therapy such as Chinese herbal medicine (CHM), manual acupuncture (MA), auricular acupuncture (AA), Qigong therapy (QT), cupping therapy (CT), scraping therapy (ST), and moxibustion (Mox). The control group was treated with any of the described interventions compared to each other or to western medicine (WM).

#### 2.1.4 Types of outcomes

The main outcome considered was the total effective rate. The secondary measure was the mean change in the Yale Global Tic Severity Scale (YGTSS) total score, YGTSS motor tics score, and YGTSS vocal tics score from the baseline. The safety outcome considered was the adverse reaction rate.

### 2.2 Exclusion criteria

The following criteria were applied to exclude studies from the analysis: studies with unclear descriptions of interventions, studies with incomplete data or without full text, studies without the outcomes specified in our NMA, and studies involving PM combined with more than one type of other TCM therapies.

#### 2.2.1 Search strategy

The search strategy for our NMA was conducted according to the PRISMA 2020 statement. Two researchers (Jiayue Liu and Hanyu Zhang) systematically searched eight databases from their inception to June, 2024, including PubMed, Cochrane Library, Web of Science, Chinese National Knowledge Infrastructure (CNKI), Chinese Biomedical Literature Database (CBM), VIP Database, and Wanfang Database. There were no regional or language restrictions. The following terms were searched: Tourette syndrome, Tic disorder, Tourette disease, multiple tics-coprolalia syndrome, pediatric massage, pediatric tuina, tuina, massage, manual therapy, randomized controlled trials, chiropractic, and RCT. Table 1 shows the search strategy of PubMed specifically, and the retrieval modes of other databases were similar.

**Table 1:** The search strategy of pubMed.

The following search strategy was employed for PubMed research:
#1 (Tourette syndrome):mh OR (Tourette syndrome):ab,ti OR (tic):ab,ti OR (Tourette disease*):ab,ti OR (Tourette disorder*):ab,ti OR (Tourette dyndrome*):ab,ti OR (chronic motor and vocal tic disorder):ab,ti OR (combined multiple motor and vocal tic disorder):ab,ti OR (combined vocal and multiple motor tic disorder):ab,ti OR (multiple motor and vocal tic disorder, combined):ab,ti OR (multiple tics-coprolalia syndrome):ab,ti OR (le Gilles de Tourette):ab,ti
#2 (massage):mh OR (massage):ab,ti OR (pediatric massage):ab,ti OR (pediatric tuina):ab,ti OR (tuina):ab,ti OR (manual therapy):ab,ti OR (chiropractic):ab,ti OR (cheirapsis):ab,ti OR (chirismus):ab,ti OR (chirapsia):ab,ti
#3 (randomized controlled trial):pt OR (controlled clinical trial):pt OR (randomized controlled trial):ab,ti OR (controlled clinical trial):ab,ti OR (trial):ab,ti OR (random*):ab,ti OR (RCT):ab,ti
#4 #1 AND #2 AND #3

#### 2.2.2 Search selection

Two researchers (Jiayue Liu and Hanyu Zhang) independently screened the studies according to the above eligibility and exclusion criteria. First, the retrieved studies were imported into NoteExpress software (16). After removing duplicate studies, preliminary screening was conducted using the titles and abstracts. Subsequently, full texts with titles and abstracts that met the criteria were carefully reviewed to determine their final inclusion. Any differences between the two researchers were resolved by discussion or consultation with a third researcher (Mengqian Lu).

### 2.3 Data extraction

Two researchers (Jiayue Liu and Hanyu Zhang) extracted the data independently using a standard information extraction table. Study characteristics were recorded, including the author, publication year, sample size, age, genders, and disease course. Intervention measures were also extracted, including those in the experimental and control groups and the treatment course. In addition, outcomes such as the total effective rate, YGTSS total score, YGTSS motor tics score, YGTSS vocal tics score, and adverse reaction rate were also extracted. Again any differences between the two researchers were resolved by discussion or consultation with a third researcher (Jinping Chen).

### 2.4 Risk of bias and quality assessment

Two researchers (Jiawei Sun and Yingqi Zhang) used the risk of bias assessment tool from the Cochrane Handbook to evaluate the quality of the included studies. The contents included random sequence generation, allocation concealment, blinding of subjects and researchers, blinding of outcome assessors, completeness of outcome data, and selective reporting of results, as well as other biases. The evaluation results included three levels: low risk, high risk, and unclear risk. Revman 5.4 software was used to make the risk of bias graph (17). The modified Jadad scale was used to assess the quality of the included studies, including the generation of randomized sequence, allocation concealment, blinding and withdrawal. With a total score of 7 on the modified Jadad scale, RCTs with a score between 1 and 3 were considered low quality, whereas studies with a score between 4 and 7 were considered high quality (18). In case of a disagreement, the two researchers negotiated or discussed with a third researcher to reach a resolution (Mengqian Lu).

### 2.5 Statistical analysis

Data synthesis was performed using Stata 17.0 software (19). A random effect model was used for all indicators. Count data were described by relative risk (RR) and 95% confidence interval (95% CI), and measurement data were described by weighted mean difference (MD) and 95% CI. The difference between the comparison groups was considered statistically significant if the 95% CI of the MD did not include 0, or the 95% CI of RR did not include 1. Additionally, the point division method was used to test the inconsistency between the direct and indirect comparison results (20). If *P* > 0.05, the consistency between them was deemed to be acceptable, and the consistency model was used for fitting. Otherwise, there was deemed to be an obvious inconsistency, and the inconsistency model was used for fitting. Subgroup analysis was used to explore the sources of inconsistency.

Probability ranking was performed using the surface under the cumulative ranking curve (SUCRA). SUCRA was expressed as a percentage, and the higher the percentage value, the better the efficacy or safety of the intervention. Sensitivity analysis was performed to evaluate the stability of the NMA results. If more than 10 studies were included, the risk of publication bias and the small sample effect were evaluated by funnel plot (21). If the image was roughly symmetric and most of the scatter points were distributed in the inverted funnel plot, it indicated that the results had less risk of publication bias and a small sample effect.

### 2.6 GRADE evaluation

The Grading of Recommendations, Assessment, Development and Evaluation (GRADE) guideline development tool was used to evaluate the quality of evidence (22). The factors that may reduce the quality of evidence include limitations in the included studies, imprecision, inconsistency, indirect evidence, and publication bias. The quality of evidence was evaluated as high, medium, low, and very low.

## 3 Results

### 3.1 Selection and identification of studies

**Figure 1:**
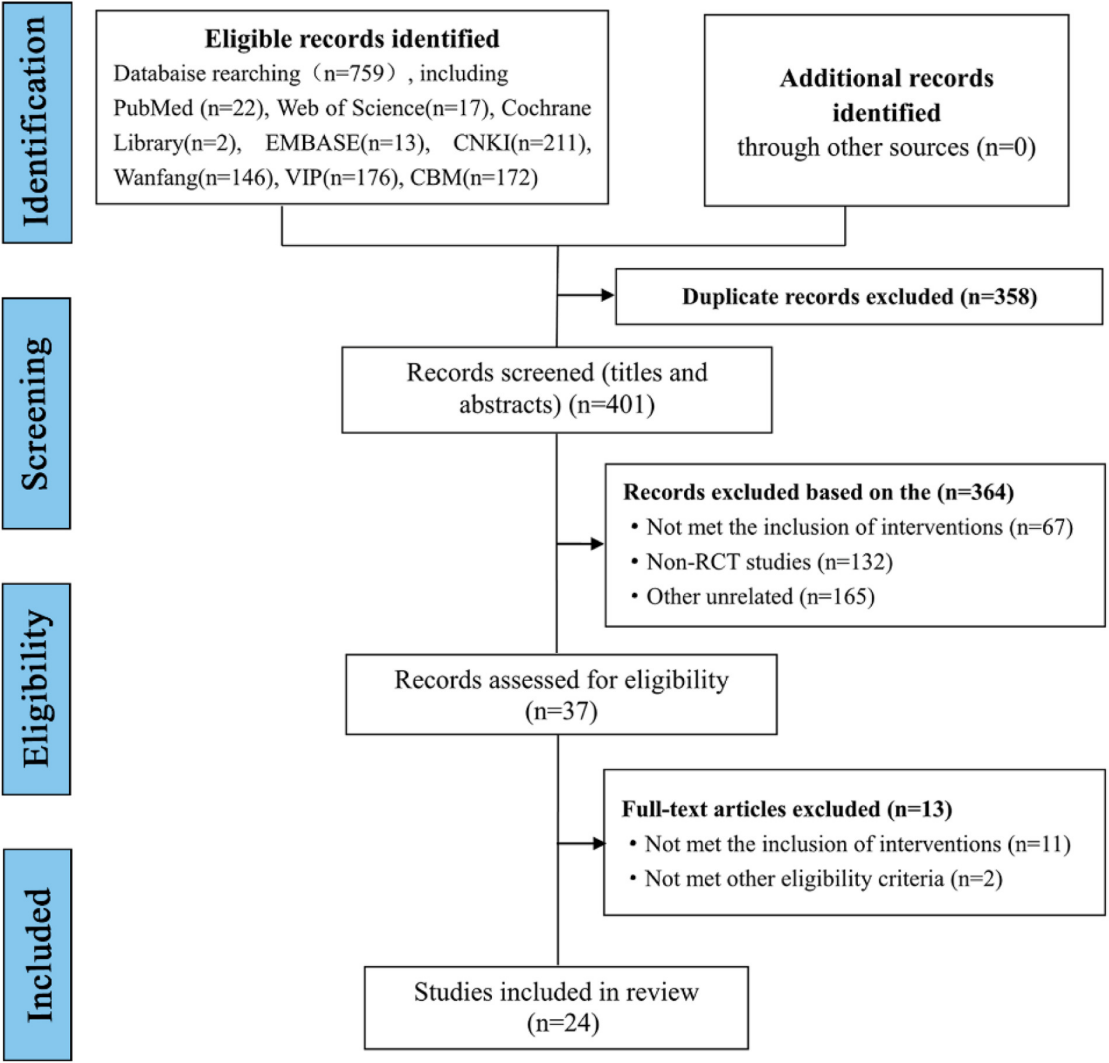
Studies search results and flow chart.

We initially retrieved 759 studies in eight databases based on the above search strategy. A total of 358 duplicate studies were excluded, and after reviewing abstracts and titles, 37 studies were obtained. Finally, a total of 24 RCTs were included by carefully reading the full texts (Figure 1).

### 3.2 Included study characteristics

A total of 24 RCTs (23–46) were included in this NMA, including 1,657 children with TD. All RCTs were conducted in China and published between 2008 and 2023. There were 9 intervention measures: pediatric massage (PM), western medicine (WM), pediatric massage (PM) + Chinese herbal medicine (CHM), pediatric massage (PM) + manual acupuncture (MA), pediatric massage (PM) + auricular acupuncture (AA), pediatric massage (PM) + Qigong therapy (QT), pediatric massage (PM) + cupping therapy (CT), pediatric massage (PM) + scraping therapy (ST), and pediatric massage (PM) + moxibustion (Mox). The characteristics of the included studies are detailed in Table 2.

**Table 2:** Characteristics of the included studies.

Study ID	Sample (E/C)	Gender (M/F)	Age (year, E/C)	Duration of illness (E/C)	Experimental group	Control group	Treatment duration	Outcomes	Modified Jadad score
Li et al. (2019) (23)	30/30	43/17	9.39 ± 2.11	(1–5)years	WM	PM + CHM	8 weeks	①②	4
Zhao (2022) (24)	36/36	51/21	(8.00 ± 2.50)/(7.75 ± 2.31)	(2.15 ± 0.56)/(1.89 ± 0.65)years	WM	PM + AA	8 weeks	①⑤	3
Liu et al. (2021) (25)	30/30	41/19	(7.07 ± 1.91)/(7.00 ± 1.66)	(8.83 ± 2.85)/(8.87 ± 2.60)months	WM	PM	2 months	①②③④	3
Xu (2019) (26)	36/36	41/31	(12.61 ± 0.59)/(12.59 ± 0.52)	(20.29 ± 2.39)months	PM	PM + CHM	3 months	①②	3
Lu (2011) (27)	105/32	107/30	(7.90 ± 2.71)/(8.28 ± 1.72)	(2.20 ± 1.05)/(2.07 ± 1.12)years	WM	PM + CHM	3 months	①	2
He (2020a) (28)	32/32	43/21	(7.39 ± 0.13)/(7.57 ± 0.30)	(1.90 ± 0.46)/(1.67 ± 0.89)years	PM	PM + MA	8 weeks	①②	3
He (2019) (29)	32/32	49/15	5–10	[4.00 (3.00, 6.75)]/[4.00 (2.25, 6.75)]months	WM	PM	12 weeks	①②③④	7
Zou et al. (2023) (30)	32/32	49/15	(7.41 ± 2.01)/(8.00 ± 2.06)	NR	WM	PM	14 days	①②⑤	4
Shi et al. (2021) (31)	45/45	54/36	(6.12 ± 1.73)/(6.35 ± 1.98)	(11.84 ± 1.54)/(12.35 ± 1.86)months	PM	PM + MA	30 days	①③④	3
Chen et al. (2022) (32)	31/27	51/7	(8.94 ± 2.35)/(8.00 ± 2.24)	NR	PM	PM + QT	5 weeks	①②③④⑤	2
Cai et al. (2019) (33)	30/30	41/19	(4.12 ± 3.53)/(4.39 ± 3.14)	(1.06 ± 0.45)/(1.11 ± 0.36)years	WM	PM + CT	3 weeks	①②	3
Wei 2009 (34)	30/30	49/11	4–16	NR	WM	PM + CHM	12 weeks	①⑤	1
Geng 2018 (35)	32/31	52/11	(6.88 ± 2.11)/(7.52 ± 2.51)	(1.33 ± 1.12)/(1.29 ± 1.10)years	WM	PM + ST	8 weeks	①②	1
Sun (2010) (36)	30/30	44/16	4–15	(3weeks–4.5years)/(2weeks–5years)	PM	PM + CHM	60 days	①	3
Zeng et al. (2016) (37)	30/30	29/31	(4.87 ± 0.78)/(4.80 ± 0.76)	(53.5 ± 9.27)/(51.67 ± 7.65)days	WM	PM + MA	10 weeks	①	3
He 2020b (38)	35/35	38/32	(10.46 ± 1.34)/(9.63 ± 1.05)	(2.66 ± 0.11)/(3.23 ± 0.14)years	WM	PM + Mox	4 weeks	①	2
Du and Wang (2016) (39)	36/36	39/33	(8.0 ± 3.8)/(7.9 ± 3.9)	(18.9 ± 7.5)/ (18.7 ± 6.5)	WM	PM + CHM	60 days	①⑤	1
Wang and Lou (2020) (40)	30/30	41/19	(7.42 ± 3.17)/(7.42 ± 3.17)	NR	PM	PM + AA	1 months	①	2
He et al. (2015) (41)	40/40	51/29	5–15	(1.20 ± 1.87)/(1.40 ± 0.78)years	WM	PM	30 days	①②	2
Li 2023 (42)	29/30	34/25	(7.93 ± 1.79)/(8.20 ± 1.99)	(1.87 ± 0.60)/(1.83 ± 0.60)years	WM	PM	8 weeks	①②③④⑤	2
Mo (2011) (43)	30/30	46/14	1–18	NR	WM	PM + CHM	2 months	①⑤	2
Du et al. (2018) (44)	42/43	45/40	(9.75 ± 0.72)/(9.28 ± 0.93)	(1.65 ± 1.20)/(1.58 ± 1.45)years	WM	PM	30 days	①②③④⑤	4
Jiang (2009) (45)	40/30	45/25	(7–15)/(6–15)	(1months–2 years)/(1days–2 years)	WM	PM + MA	60 days	①	2
Chen and Mi (2008) (46)	29/28	39/18	(5.23 ± 1.09)/(4.93 ± 0.97)	(0.62 ± 0.13)/(0.59 ± 0.24)years	WM	PM	20 days	①⑤	4

① The total effective rate; ② the Yale global tic severity scale (YGTSS) total score; ③ the YGTSS motor tics score; ④ the YGTSS vocal tics score; ⑤ the adverse reaction rate. E, experimental group; C, control group; M, male; F, female; NR, not reported; WM, western medicine; PM, pediatric massage; CHM, Chinese herbal medicine; MA, manual acupuncture; AA, auricular acupuncture; QT, qigong therapy; CT, cupping therapy; ST, scraping therapy; Mox, moxibustion.

### 3.3 Methodological quality assessment

**Figure 2:**
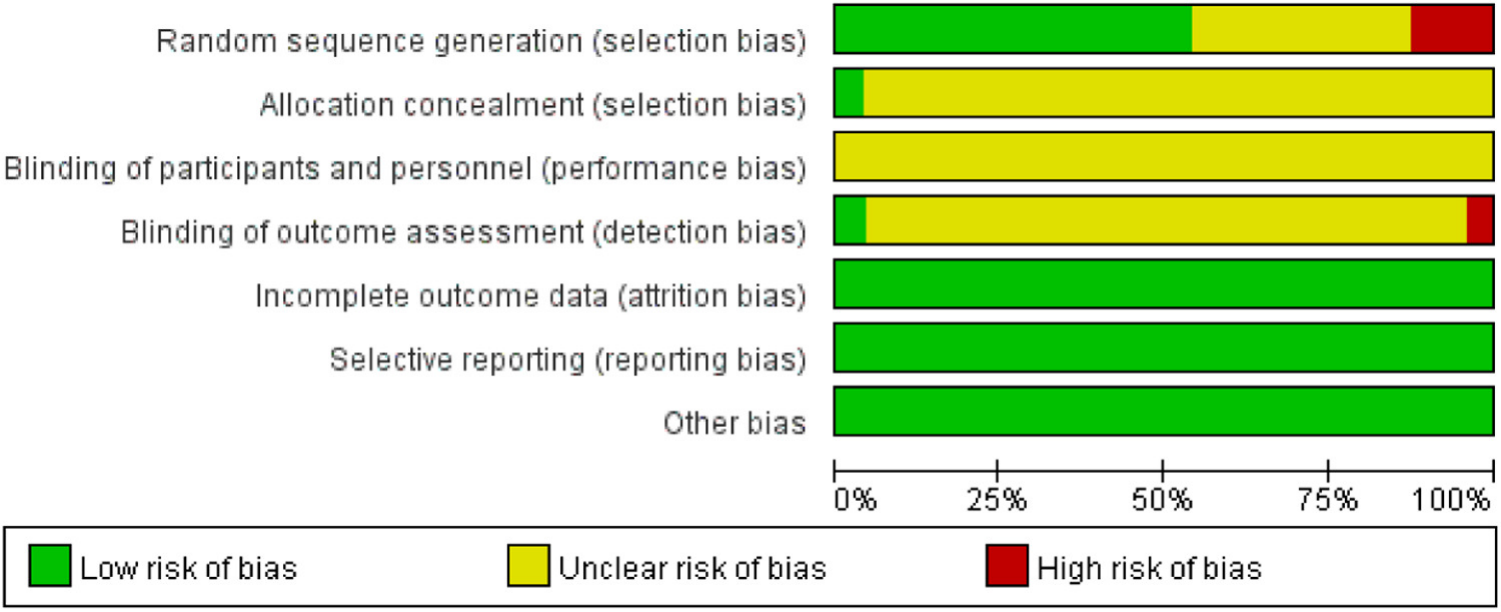
Bias risk assessment.

**Figure 3:**
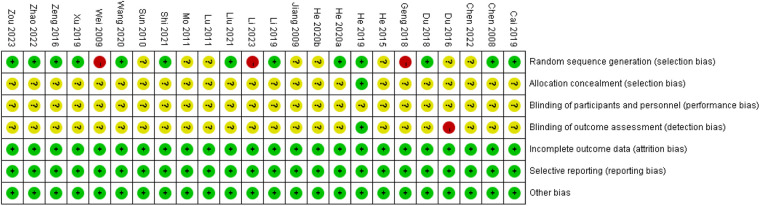
Risk of bias summary.

The results of the methodological quality assessment are shown in Figures 2, 3. Twelve studies (23–25, 28–31, 33, 37, 40, 44, 46) used the random number table method for randomization, and one study^18^ used the lottery method. Eight studies (27, 32, 36, 38, 39, 41, 43, 45) referred only to “randomization” without describing the method. Three studies (35, 42) used randomization methods that were considered high risk. One study (29) was rated as low risk for blinding of outcome assessment. One study (29) used the envelope method for allocation concealment, and the remaining studies did not mention specific allocation concealment methods. All the studies were considered low risk for data completeness and selective reporting. Five studies (23, 29, 30, 44, 46) were identified as high quality and the remaining (24–28, 31–43, 45) as low quality based on the modified Jadad score.

### 3.4 Network plots

**Figure 4:**
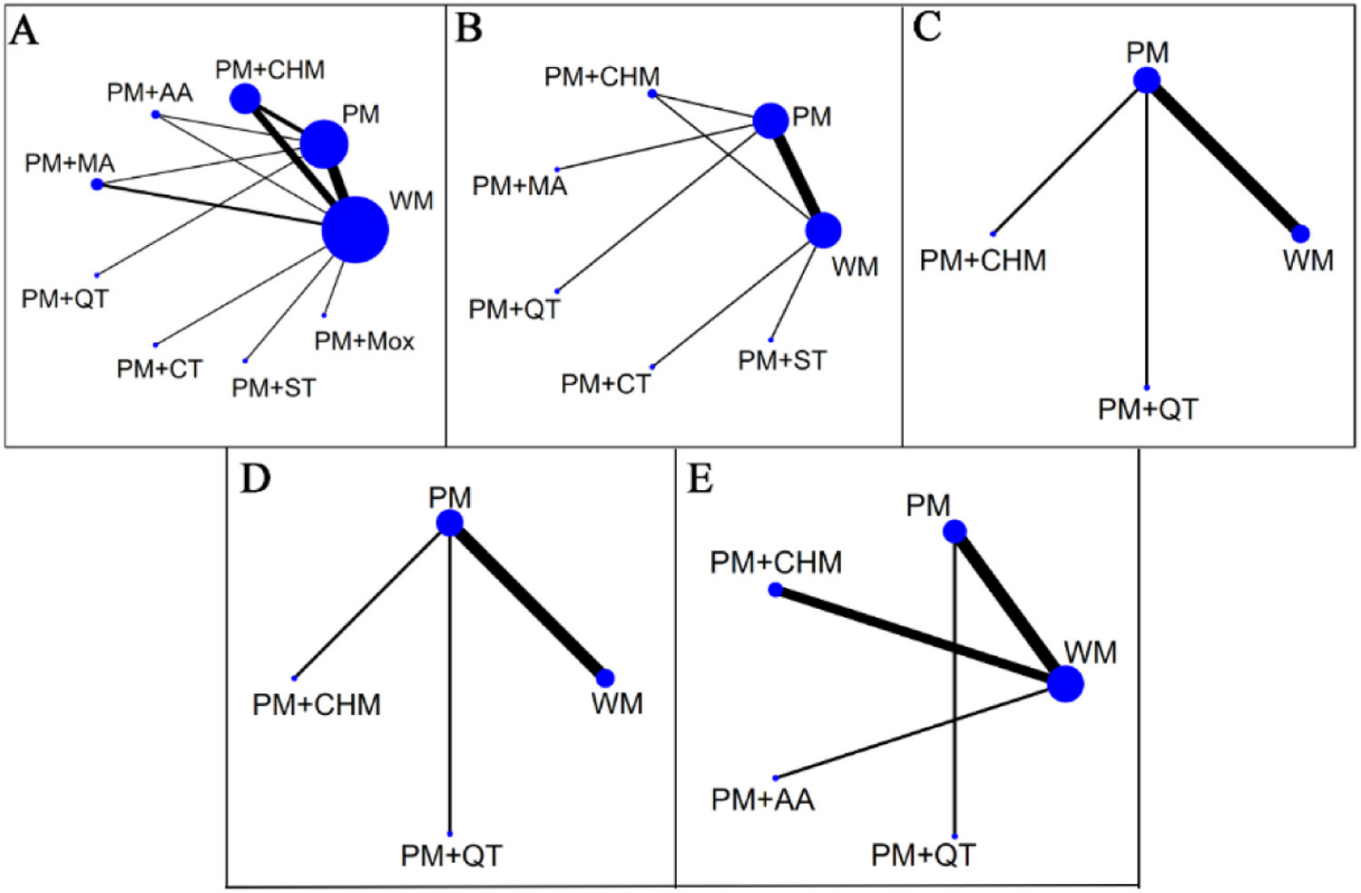
Network plots. Network plots for the total effective rate **(A)**, the YGTSS total score **(B)**, the YGTSS motor tics score **(C)**, the YGTSS vocal tics score **(D)**, and adverse reaction rate **(E)** Blue circles represent the number of patients who received the therapy, and lines represent the presence of direct comparative evidence between the two therapies. WM, western medicine; PM, pediatric massage; CHM, Chinese herbal medicine; MA, manual acupuncture; AA, auricular acupuncture; QT, qigong therapy; CT, cupping therapy; ST, scraping therapy; Mox, moxibustion.

The evidence relationships of the included studies are shown in Figure 4. In the network plots, the blue circles represent the number of studies involving the intervention, and the lines represent the number of studies that directly compare the two interventions. Regarding the total effective rate, 24 studies (23–46) were included in the NMA, involving 1,657 patients and 9 interventions. Regarding the YGTSS total score, 12 studies (23, 25, 26, 28–30, 32, 33, 35, 38, 42, 44) were amalgamated for the NMA, involving 843 patients and 7 interventions. Six studies (22, 25, 29, 32, 42, 44) reported YGTSS motor tic scores and vocal tic scores, including 416 patients and four interventions. With respect to the adverse reaction rate, 9 studies (24, 30, 32, 34, 39, 42–44, 46) were amalgamated for the NMA that covered 587 patients and 5 interventions.

### 3.5 Inconsistency test

The results of our inconsistency test indicated that in terms of total clinical effective rate, there was inconsistency between the direct and indirect comparison results of the closed-loop “western medicine (WM)—pediatric massage (PM)- pediatric massage (PM) + manual acupuncture (MA)” (*P* = 0.038). Therefore, this index was fitted using the inconsistent model, and the indirect comparison results need to be interpreted carefully. The results of the direct and indirect comparison of the YGTSS total score were consistent (*P* > 0.05), and the consistency model was used to fit. Since the evidence relationship for the YGTSS motor tics score, YGTSS vocal tics score, and adverse reaction rate did not form a closed loop, there was no need for an inconsistency test.

### 3.6 NMA results

#### 3.6.1 Total effective rate

**Figure 5:**
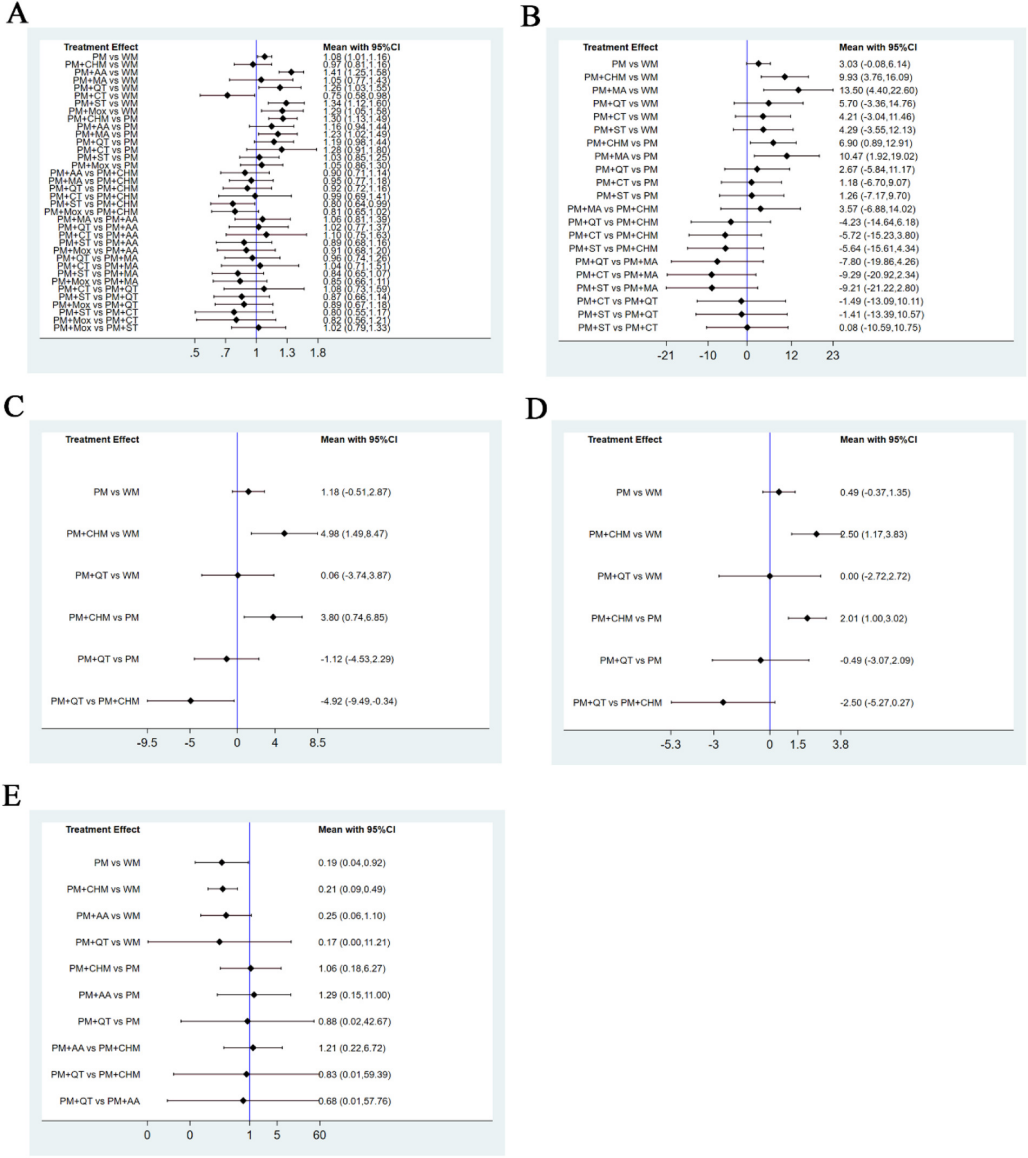
Forest plots for pairwise comparisons. Forest plots for pairwise comparisons of the total effective rate **(A)**, the YGTSS total score **(B)**, the YGTSS motor tics score **(C)**, the YGTSS vocal tics score **(D)**, and adverse reaction rate **(E)**.

Compared to western medicine (WM), pediatric massage (PM), pediatric massage (PM) + auricular acupuncture (AA), pediatric massage (PM) + Qigong therapy (QT), pediatric massage (PM) + moxibustion (Mox), and pediatric massage (PM) + scraping therapy (ST) each significantly increased the total effective rate. Moreover, compared to simple pediatric massage (PM), pediatric massage (PM) + Chinese herbal medicine (CHM) and pediatric massage (PM) + manual acupuncture (MA) significantly increased the total effective rate. In addition, compared to pediatric massage (PM) + scraping therapy (ST), pediatric massage (PM) + Chinese herbal medicine (CHM) had greater effects on the total effective rate. The 95%CI of the above RR values did not include 1, so the differences were considered to be statistically significant. There were no statistically significant differences between any other pairwise comparisons (Table 3; Figure 5A).

**Table 3:** Results of the NMA for the total effective rate.

The total effective rate								
**PM** **+** **CHM**								
1.01 (0.71, 1.44)	**PM** **+** **CT**							
1.05 (0.85, 1.30)	1.04 (0.71, 1.51)	**PM** **+** **MA**						
1.09 (0.86, 1.38)	1.08 (0.73, 1.59)	1.04 (0.79, 1.36)	**PM** **+** **QT**					
1.12 (0.88, 1.42)	1.10 (0.75, 1.63)	1.06 (0.81, 1.39)	1.02 (0.77, 1.37)	**PM** **+** **AA**				
1.23 (0.98, 1.55)	1.22 (0.83, 1.79)	1.17 (0.90, 1.52)	1.13 (0.85, 1.49)	1.10 (0.83, 1.46)	**PM** **+** **Mox**			
1.26 (1.01, 1.56)*	1.24 (0.85, 1.81)	1.20 (0.93, 1.54)	1.15 (0.88, 1.51)	1.13 (0.86, 1.48)	1.02 (0.79, 1.33)	**PM** **+** **ST**		
1.30 (1.13, 1.49)*	1.28 (0.91, 1.80)	1.23 (1.02, 1.49)*	1.19 (0.98, 1.44)	1.16 (0.94, 1.44)	1.05 (0.86, 1.30)	1.03 (0.85, 1.25)	**PM**	
0.97 (0.81, 1.16)	0.75 (0.58, 0.98)	1.05 (0.77, 1.43)	1.26 (1.03, 1.55)*	1.41 (1.25, 1.58)*	1.29 (1.05, 1.58)*	1.34 (1.12, 1.60)*	1.08 (1.01, 1.16)*	**WM**

* Indicates comparisons with statistically significant differences (*P* < 0.05). WM, western medicine; PM, pediatric massage; CHM, Chinese herbal medicine; MA, manual acupuncture; AA, auricular acupuncture; QT, qigong therapy; CT, cupping therapy; ST, scraping therapy; Mox, moxibustion.

**Figure 6:**
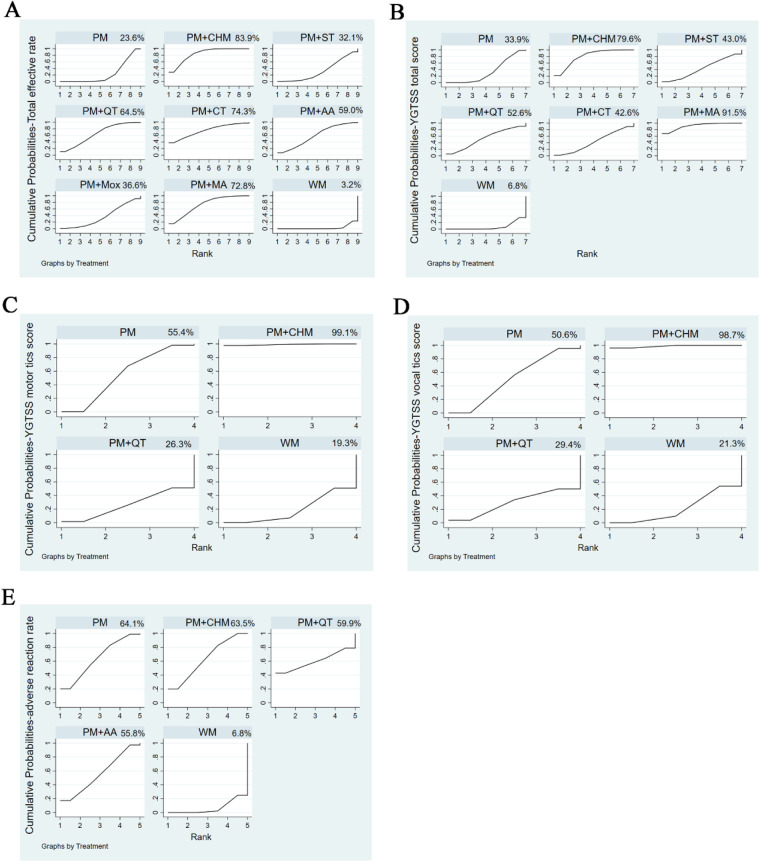
SUCRA values. SUCRA values for the total effective rate **(A)**, the YGTSS total score **(B)**, the YGTSS motor tics score **(C)**, the YGTSS vocal tics score **(D)**, and adverse reaction rate **(E)** The higher the SUCRA value, the better the efficacy or safety of the intervention.

According to the SUCRA values (Figure 6A), the probability of interventions in descending order was as follows: pediatric massage (PM) + Chinese herbal medicine (CHM), pediatric massage (PM) + cupping therapy (CT), pediatric massage (PM) + manual acupuncture (MA), pediatric massage (PM) + Qigong therapy (QT), pediatric massage (PM) + auricular acupuncture (AA), pediatric massage (PM) + moxibustion (Mox), pediatric massage (PM) + scraping therapy (ST), simple pediatric massage (PM), western medicine (WM).

#### 3.6.2 YGTSS total score

Compared to western medicine (WM) and simple pediatric massage (PM), pediatric massage (PM) + Chinese herbal medicine (CHM) and pediatric massage (PM) + manual acupuncture (MA) significantly decreased the YGTSS total score. The 95% CI of MD values did not include 0, so the above differences were judged to be statistically significant. There were no statistically significant differences between any other pairwise comparisons (Table 4; Figure 5B).

**Table 4:** Results of the NMA for the YGTSS total score.

YGTSS total score						
**PM** **+** **MA**						
3.57 (−6.88, 14.02)	**PM** **+** **CHM**					
7.80 (−4.26, 19.86)	4.23 (−6.18, 14.64)	**PM** **+** **QT**				
9.21 (−2.80, 21.22)	5.64 (−4.34, 15.61)	1.41 (−10.57, 13.39)	**PM** **+** **ST**			
9.29 (−2.34, 20.92)	5.72 (−3.80, 15.23)	1.49 (−10.11, 13.09)	0.08 (−10.59, 10.75)	**PM** **+** **CT**		
10.47 (1.92, 19.02)*	6.90 (0.89, 12.91)*	2.67 (−5.84,1 1.17)	1.26 (−7.17, 9.70)	1.18 (−6.70, 9.07)	**PM**	
13.50 (4.40, 22.60)*	9.93 (3.76, 16.09)*	5.70 (−3.36, 14.76)	4.29 (−3.55, 12.13)	4.21 (−3.04, 11.46)	3.03 (−0.08, 6.14)	**WM**

* Indicates comparisons with statistically significant differences (*P* < 0.05). WM, western medicine; PM, pediatric massage; CHM, Chinese herbal medicine; MA, manual acupuncture; QT, qigong therapy; CT, cupping therapy; ST, scraping therapy.

According to the SUCRA values (Figure 6B), the probability of decreasing the YGTSS total score in descending order was as follows: pediatric massage (PM) + manual acupuncture (MA), pediatric massage (PM) + Chinese herbal medicine (CHM), pediatric massage (PM) + Qigong therapy (QT), pediatric massage (PM) + scraping therapy (ST), pediatric massage (PM) + cupping therapy (CT), simple pediatric massage (PM), western medicine (WM).

#### 3.6.3 YGTSS motor tics score

Compared to western medicine (WM), simple pediatric massage (PM), and pediatric massage (PM) + Qigong therapy (QT), pediatric massage (PM) + Chinese herbal medicine (CHM) significantly reduced the YGTSS motor tics score. The 95% CI of MD values did not include 0, so the above differences were statistically significant. There were no statistically significant differences between any other pairwise comparisons (Table 5; Figure 5C).

**Table 5:** Results of the NMA for the YGTSS motor tics score.

YGTSS motor tics score			
**PM** **+** **CHM**			
3.80 (0.74, 6.85)*	**PM**		
4.92 (0.34, 9.49)*	1.12 (−2.29, 4.53)	**PM** **+** **QT**	
4.98 (1.49, 8.47)*	1.18 (−0.51, 2.87)	0.06 (−3.74, 3.87)	**WM**

* Indicates comparisons with statistically significant differences (*P* < 0.05). WM, western medicine; PM, pediatric massage; CHM, Chinese herbal medicine; QT, qigong therapy.

According to the SUCRA values (Figure 6C), the probability for decreasing the YGTSS score in descending order was as follows: pediatric massage (PM) + Chinese herbal medicine (CHM), simple pediatric massage (PM), pediatric massage (PM) + Qigong therapy (QT), western medicine (WM).

#### 3.6.4 YGTSS vocal tics score

Compared to western medicine (WM) and simple pediatric massage (PM), pediatric massage (PM) + Chinese herbal medicine (CHM) significantly decreased the YGTSS vocal tics score. The 95%CI of MD values did not include zero, so the above differences were statistically significant. There were no statistically significant differences between any other pairwise comparisons (Table 6; Figure 5D).

**Table 6:** Results of the NMA for the YGTSS vocal tics score.

YGTSS vocal tics score			
**PM** **+** **CHM**			
2.01 (1.00, 3.02)*	**PM**		
2.50 (−0.27, 5.27)	0.49 (−2.09, 3.07)	**PM** **+** **QT**	
2.50 (1.17, 3.83)*	0.49 (−0.37, 1.35)	0.00 (−2.72, 2.72)	**WM**

* Indicates comparisons with statistically significant differences (*P* < 0.05). WM, western medicine; PM, pediatric massage; CHM, Chinese herbal medicine; QT, qigong therapy.

According to the SUCRA values (Figure 6D), the probability for decreasing the YGTSS score in descending order was as follows: pediatric massage (PM) + Chinese herbal medicine (CHM), simple pediatric massage (PM), pediatric massage (PM) + Qigong therapy (QT), western medicine (WM).

#### 3.6.5 Adverse reaction rate

Compared to western medicine (WM), simple pediatric massage (PM) and pediatric massage (PM) + Chinese herbal medicine (CHM) significantly reduced the adverse reaction rate. Here again the 95% CI of RR values did not include 1, so the above differences were deemed to be statistically significant. Again, there were no statistically significant differences between any other pairwise comparisons (Table 7; Figure 5E). According to the SUCRA values (Figure 6E), the probability of reducing the adverse reaction rate in descending order was as follows: simple pediatric massage (PM), pediatric massage (PM) + Chinese herbal medicine (CHM), pediatric massage (PM) + Qigong therapy (QT), pediatric massage (PM) + auricular acupuncture (AA), western medicine (WM).

**Table 7:** Results of the NMA for the adverse reaction rate.

The adverse reaction rate				
**PM**				
0.94 (0.16, 5.59)	**PM** **+** **CHM**			
1.14 (0.02, 55.72)	1.21 (0.02, 86.91)	**PM** **+** **QT**		
0.78 (0.09, 6.66)	0.82 (0.15, 4.56)	0.68 (0.01, 57.76)	**PM** **+** **AA**	
0.19 (0.04, 0.92)*	0.21 (0.09, 0.49)*	0.17 (0.00, 11.21)	0.25 (0.06, 1.10)	**WM**

* Indicates comparisons with statistically significant differences (*P* < 0.05). WM, western medicine; PM, pediatric massage; CHM, Chinese herbal medicine; AA, auricular acupuncture; QT, qigong therapy.

Pediatric massage (PM) + auricular acupuncture (AA) may cause fatigue and lethargy, and pediatric massage (PM) + Chinese herbal medicine (CHM) may cause gastrointestinal symptoms such as nausea (24, 34, 39, 43). These effects were considered to be relatively mild and acceptable. Western medicine (WM) may lead to adverse reactions such as dizziness, somnolence, diarrhea, rash, and tachycardia (24, 30, 34, 39, 42–44, 46). No obvious adverse reactions were reported with simple pediatric massage (PM) or pediatric massage (PM) + Qigong therapy (QT) (30, 32, 43, 44, 46).

#### 3.6.6 Evaluation of stability of results

**Figure 7:**
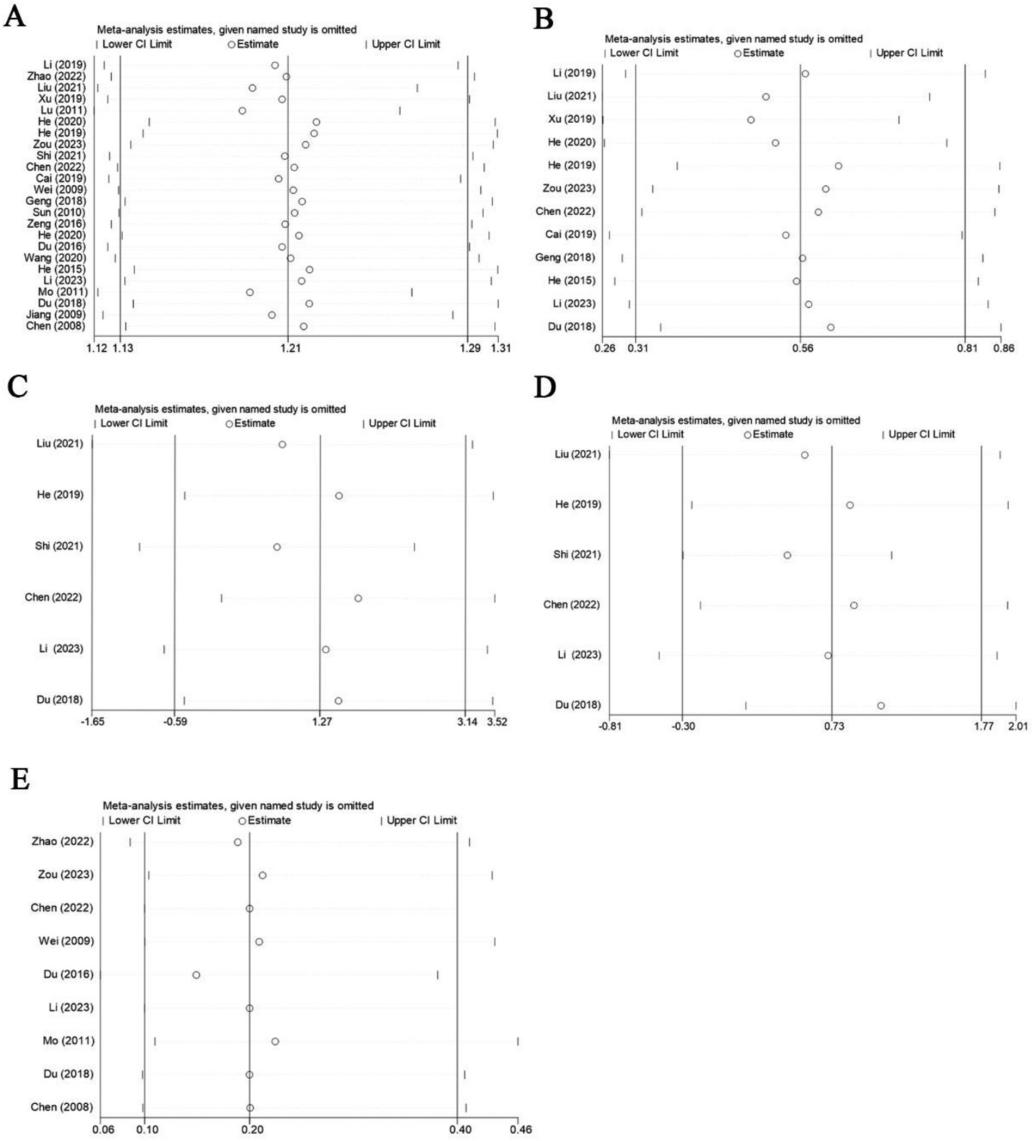
Sensitivity analysis diagrams. Sensitivity analysis for the total effective rate **(A)**, the YGTSS total score **(B)**, the YGTSS motor tics score **(C)**, the YGTSS vocal tics score **(D)**, and adverse reaction rate **(E)**.

To assess the stability of the findings, we performed sensitivity analyses (Figure 7) and recalculated core outcome measures after excluding studies at high risk of bias (24–28, 31–43, 45). Reanalysis after excluding studies with high risk of bias showed that PM + CHM was most likely to have the best clinical response rate, which was consistent with the results before exclusion (Supplementary Material 1A–C). However, the therapies that ranked first in the total YGTSS score were different from those before exclusion (Supplementary Material 1D, E). This is presumably due to the fact that the original top-ranked therapy was not included in the remaining low-bias studies. This difference in results suggests that a higher proportion of the original studies at high risk of bias may have had an effect on the stability of some outcomes.

#### 3.6.7 Publication bias and small sample effect

**Figure 8:**
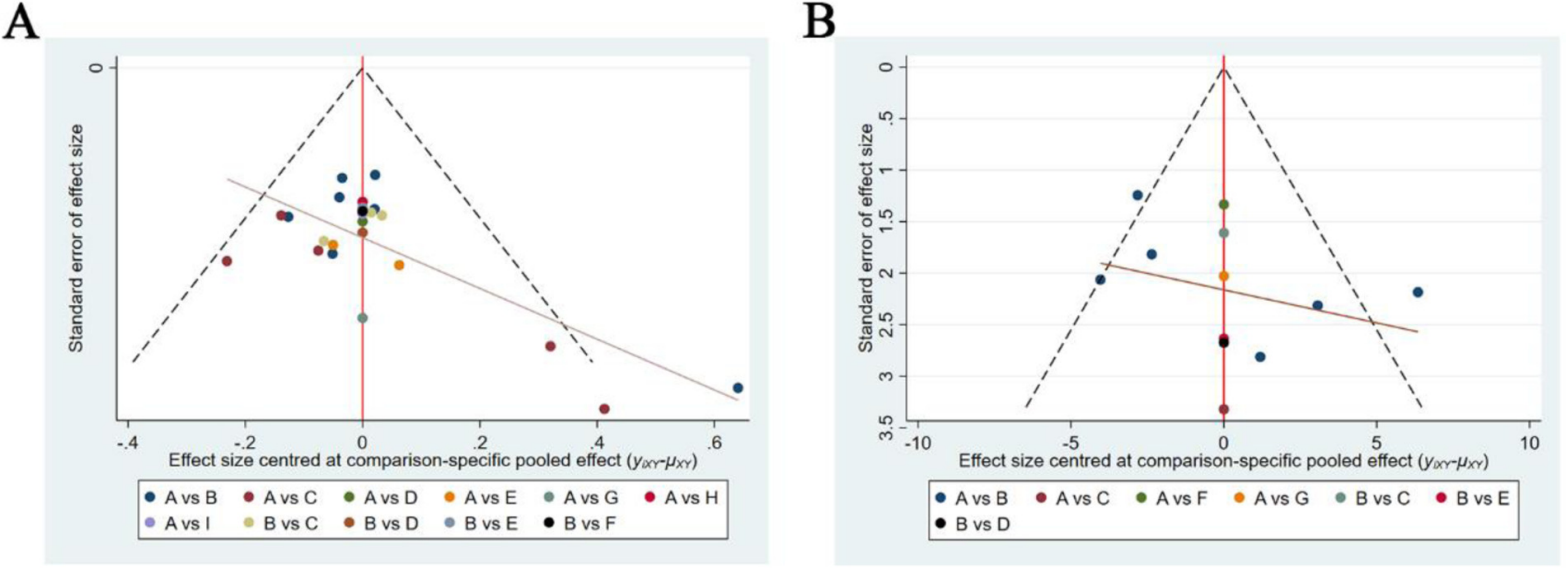
Comparison-correction funnel plots. Comparison-adjusted funnel plots for the total effective rate **(A)** and YGTSS score **(B)**. If the image was roughly symmetric and most of the scatter points were distributed in the inverted funnel plot, the results had less risk of publication bias and a small sample effect.

Comparison-correction funnel plots are shown in Figure 8. The comparison-corrected funnel plot has a roughly symmetrical distribution, though some scattered points were outside the funnel plot, suggesting the possibility of moderate publication bias or small sample effects. Fewer than 10 studies reported adverse reaction rates, so the comparison-corrected funnel plot for this index was not analyzed.

### 3.7 Results of GRADE evaluation

Results of GRADE evaluation are shown in Table 8. The evidence quality for the total effective rate was rated as “very low”, while the evidence quality for the YGTSS total score was rated as “low”. The evidence quality for the YGTSS vocal tic score, YGTSS motor tic score, and the adverse reaction rate was rated as “moderate”.

**Table 8:** Results of GRADE evaluation.

Outcomes	Included studies’ limitations	Imprecision	Inconsistency	Indirect evidence	Publication bias	Quality of evidence
Total effective rate	−1	0	−1	0	−1	Very low
YGTSS total score	−1	0	0	0	−1	Low
YGTSS motor tics score	−1	0	0	0	0	Moderate
YGTSS vocal tics score	−1	0	0	0	0	Moderate
Adverse reaction rate	−1	0	0	0	0	Moderate

″−1″ indicates a downgrade by one level, while ″0″ indicates no downgrade. The quality of evidence for each outcome is categorized as “High” if no downgrading is applied, “Moderate” if downgraded by one level, “Low” if downgraded by two levels, and “Very Low” if downgraded by three or more levels.

### 3.8 Subgroup analysis

**Figure 9:**
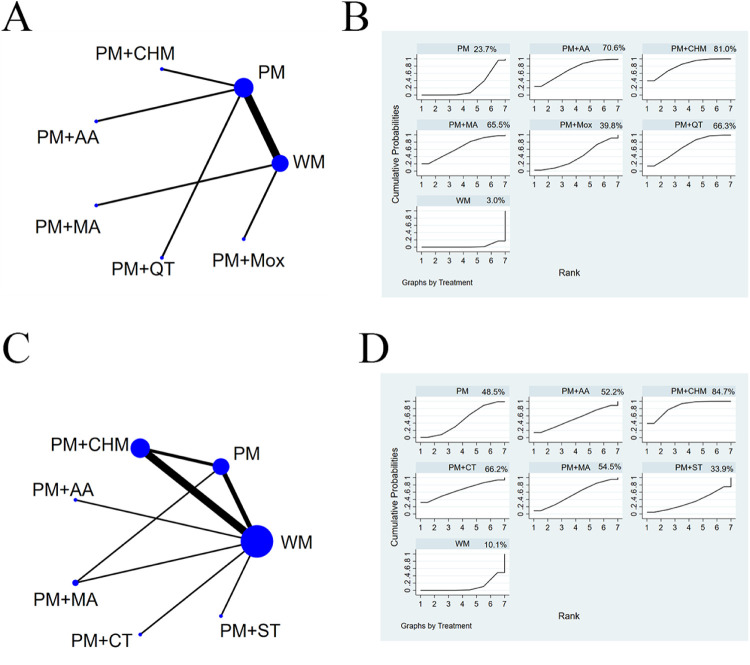
Results of the subgroup analysis for the total effective rate. Network plots for the total effective rate in the subgroups of treatment course <2months **(A)** and treatment course ≥2months **(C)** SUCRA values for the total effective rate in the subgroups of treatment course <2months **(B)** and treatment course ≥2months **(D)** WM, western medicine; PM, pediatric massage; CHM, Chinese herbal medicine; MA, manual acupuncture; AA, auricular acupuncture; QT, qigong therapy; CT, cupping therapy; ST, scraping therapy; Mox, moxibustion.

**Figure 10:**
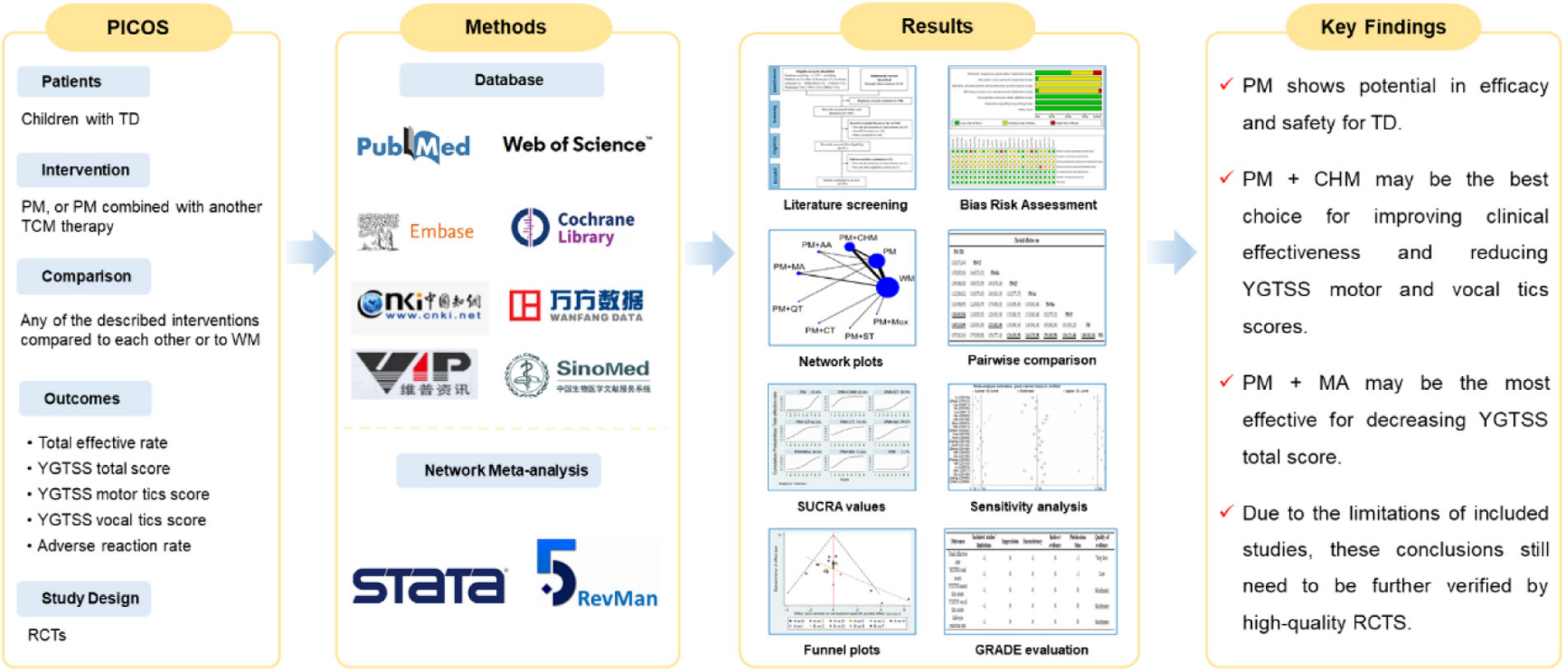
Graphical abstract. TD, tic disorders; PM, pediatric massage; TCM, traditional Chinese medicine treatment; WM, western medicine; YGTSS, Yale global tic severity scale; RCTs, randomized controlled trials; SUCRA, surface under the cumulative ranking curve; GRADE, the grading of recommendations, assessment, development and evaluation; CHM, Chinese herbal medicine; MA, manual acupuncture.

Since there were inconsistency in the closed-loop “WM-PM-PM + MA” of total clinical effective rate, subgroup analysis was used to explore the potential causes. Considering that the differences in treatment courses may affect the research results, two subgroups were established: treatment courses ≥2 months and treatment courses <2 months. The results are detailed in Tables 9, 10; Figures 9, 10. In the subgroup with a treatment course <2 months, compared to western medicine (WM), pediatric massage (PM) + Chinese herbal medicine (CHM), pediatric massage (PM) + auricular acupuncture (AA), pediatric massage (PM) + manual acupuncture (MA), and pediatric massage (PM) + Qigong therapy (QT) each significantly increased the total effective rate (*P* < 0.05). Moreover, compared to simple pediatric massage (PM), pediatric massage (PM) + Chinese herbal medicine (CHM) significantly increased the total effective rate (*P* < 0.05). In the subgroup with a treatment course ≥2 months, compared to western medicine (WM), simple pediatric massage (PM) and pediatric massage (PM) + Chinese herbal medicine (CHM) significantly increased the total effective rate (*P* < 0.05). In both subgroups, the likely best outcome in terms of total response rate was pediatric massage (PM) + Chinese herbal medicine (CHM).What's more, the results showed that there was no closed loop of evidence relationship in the subgroup of treatment duration <2 months, and there was no significant inconsistency in the closed loop of “WM-PM-PM + MA” in the subgroup of treatment duration ≥2 months (*P* > 0.05). It can be seen that the duration of treatment may account for the inconsistency between the direct and indirect evidence for the overall response rate.

**Table 9:** Results of the subgroup analysis (treatment course <2 months) for the total effective rate.

The total effective rate (treatment course <2 months)						
**PM** **+** **CHM**						
1.05 (0.78, 1.40)	**PM** **+** **AA**					
1.07 (0.82, 1.41)	1.03 (0.77, 1.37)	**PM** **+** **QT**				
1.07 (0.78, 1.47)	1.03 (0.74, 1.43)	1.00 (0.73, 1.37)	**PM** **+** **MA**			
1.20 (0.90, 1.59)	1.14 (0.84, 1.55)	1.12 (0.84, 1.48)	1.11 (0.82, 1.51)	**PM** **+** **Mox**		
1.27 (1.05, 1.54)	1.22 (0.98, 1.52)	1.19 (0.98, 1.44)	1.18 (0.92, 1.52)	1.06 (0.86, 1.31)	**PM**	
1.37 (1.11, 1.69)*	1.31 (1.03, 1.65)*	1.28 (1.04, 1.57)*	1.27 (1.01, 1.61)*	1.14 (0.94, 1.39)	1.07 (0.99, 1.17)	**WM**

* Indicates comparisons with statistically significant differences (*P* < 0.05). WM, western medicine; PM, pediatric massage; CHM, Chinese herbal medicine; AA, auricular acupuncture; QT, qigong therapy; MA, manual acupuncture; Mox, moxibustion.

**Table 10:** Results of the subgroup analysis (treatment course ≥ 2 months) for the total effective rate.

The total effective rate (treatment course ≥2 months)						
**PM** **+** **CHM**						
1.08 (0.64, 1.82)	**PM** **+** **CT**					
1.19 (0.84, 1.67)	1.10 (0.61, 1.96)	**PM** **+** **MA**				
1.19 (0.76, 1.87)	1.10 (0.58, 2.08)	1.01 (0.60, 1.69)	**PM** **+** **AA**			
1.22 (0.98, 1.51)	1.13 (0.66, 1.91)	1.03 (0.75, 1.40)	1.02 (0.65, 1.61)	**PM**		
1.34 (0.87, 2.08)	1.24 (0.66, 2.33)	1.13 (0.68, 1.88)	1.13 (0.64, 1.99)	1.10 (0.71, 1.73)	**PM** **+** **ST**	
1.50 (1.24, 1.81)*	1.39 (0.85, 2.26)	1.27 (0.92, 1.74)	1.26 (0.84, 1.90)	1.23 (1.01, 1.51)*	1.12 (0.75, 1.66)	**WM**

* Indicates comparisons with statistically significant differences (*P* < 0.05). WM, western medicine; PM, pediatric massage; CHM, Chinese herbal medicine; AA, auricular acupuncture; CT, cupping therapy; MA, manual acupuncture; ST, scraping therapy.

To clarify the impact of standardized protocols on outcomes, we evaluated the standardization degree of included RCTs based on whether PM operations adhered to expert consensus, guidelines, or textbooks, and conducted subgroup analyses according to the standardization degree of PM. The results showed that 3 RCTs (29, 30, 32) had good standardization because they performed PM operations by nationally planned textbooks (47–49). The remaining RCTs (23–28, 31, 33–46) did not mention the consensus, guidelines, or textbooks followed for PM operations (Supplementary Material 2). Subgroup analysis results indicated that in the subgroup with a higher degree of PM operation standardization, PM + QT had the highest probability of achieving the best efficacy in improving the total effective rate and reducing the total YGTSS score (Supplementary Material 3A–C, Supplementary Material 4A–C). In the subgroup with a lower degree of PM operation standardization, PM + CHM had the highest possibility of being the most effective in improving the total effective rate, while PM + MA had the highest probability of ranking first in reducing the total YGTSS score (Supplementary Material 3D–F, Supplementary Material 4D–F). This suggests that the impact of the standardization of PM operation techniques on outcomes deserves attention.

## 4 Discussion

### 4.1 The efficacy and safety of PM are positive for TD in children

PM has a significant curative effect on TD in children. The results of this NMA suggest the efficacy of PM in improving the total effective rate was significantly better than WM and that its efficacy in reducing YGTSS score was equivalent to WM. According to TCM theory, the pathogenesis of TD in children is related to spleen deficiency, liver hyperactivity, phlegm and fire disturbance, and wind formation from yin deficiency (50, 51). PM programs for TD include chiropractic, abdominal massage, and acupressure (12, 31). These PM techniques can relax the meridians, regulate the viscera, and relieve the somatic symptoms of TD (52). In addition, PM has the characteristics of being peaceful and gentle, and it can be combined with language communication to soothe the emotions of children during treatment (53). Modern medical studies have shown that PM can also improve the function of the hippocampus and other related brain regions by regulating the expression of brain-derived neurotrophic factor (BDNF), insulin-like growth factor-1 (IGF-1), 5-hydroxytryptamine 1A receptor (5-HT1AR), and synaptophysin-1 (Syn1), which enables it to treat neuropsychiatric symptoms caused by many diseases (54, 55). A previous systematic review and meta-analysis showed that PM improved clinical efficacy and reduced the YGTSS score and TCM syndrome score (56).

PM also shows potential in terms of safety for treating children with TD. The results of this NMA suggest that the probability safety in of simple PM was ranked first, and its incidence of adverse reactions was significantly lower than that of WM. Because the physiological systems of children are sensitive, the safety of therapies is particularly important for treating those with TD. PM is a non-invasive treatment with gentle and mild manipulation, which can minimize the stimulation of children's sensitive physiological system and reduce the risk of trauma (57). In addition, As a drug-free therapy, PM can avoid drug-related toxic and side effects, meeting the needs of children for treatment safety. The above intervention characteristics of non-invasive physical stimulation make PM have unique safety characteristics suitable for children. However, it should be noted that safety data in this analysis is limited, with only 9 RCTs reporting adverse reactions. Therefore, while this study provides preliminary NMA evidence supporting the safety of PM in treating children with TD, these findings should be interpreted with caution.

### 4.2 PM + CHM and PM + MA may have important advantages for TD in children

PM + CHM may be the best therapy for improving the clinically effective rate of TD. This NMA found that PM + CHM had the highest probability of enhancing the total effective rate and reducing the YGTSS motor tics score and vocal tics score. In addition, PM + CHM had the second highest probability of reducing YGTSS total score, and its efficacy was significantly better than that of simple PM. CHM plays an important role in complementary and alternative medicine for treating TD. According to TCM theory, CHM can improve the overall condition of TD patients by calming the liver, quenching the wind, resolving phlegm, and invigorating the spleen (58, 59). Modern medical research has also shown that CHM can alleviate behavioral symptoms of TD by inhibiting inflammatory responses, correcting neurotransmitter imbalances, and repairing nerve damage (60, 61). PM can relieve muscle spasms and calm the mind by stimulating local acupoints, and CHM can improve visceral functions by overall regulation. The combination of PM and CHM conforms to the principle of “internal and external treatment” in TCM. The synergistic effect of these two therapies may be the reason for their better efficacy compared to simple PM.

PM + MA also performed pretty well in relieving symptoms of TD in children. Our NMA results ranked PM + MA highest in probability of reducing the YGTSS total score and ranked in the top three in enhancing total effective rate. A previous NMA has demonstrated that acupuncture plus PM has a significant effect in improving clinical efficacy and reducing motor and vocal tics of children with TD, consistent with our findings (62). MA can regulate the circulation of qi and blood in meridians and relieve muscle spasticity so as to alleviate the convulsion symptoms of children with TD (63). Additionally, acupuncture at Baihui (GV20) and Yintang (GV29) can reduce the stereotypical behavior of Tourette's syndrome (TS) mice by reducing the expression of dopamine receptors in the striatum (STR) and substantia nigra pars compact (SNpc) (64). One study has also shown that PM + MA can maintain the balance of intestinal flora through “brain-gut axis” regulation and effectively relieve neuropsychiatric symptoms such as tic, irritability, and fatigue in children with TD (65). PM + MA may have a potential synergistic effect in relaxing muscles and regulating brain function and in doing so provide a curative effect on TD.

It can be seen that PM + CHM and PM + MA have their own mechanisms and advantages in the treatment of TD. CHM can regulate the neuroendocrine immune network, and PM can stimulate local muscles and nerves (32, 66). The combination of CHM and PM can improve the body state through internal and external interaction. This may be the reason for the outstanding efficacy of PM + CHM in the comprehensive efficacy indicators such as total effective rate. MA has a regulatory effect on the nervous system, and PM can relieve muscle spasticity (67). This may be the reason why PM + MA is more targeted to improve the total score of YGTSS, a neurological function related index. The above mechanistic differences between PM + CHM and PM + MA provide an explanation for the difference in ranking.

It is important to note that although these SUCRA rankings provide a reference for comparisons of intervention efficacy and safety, the wide confidence intervals in several comparisons reflect possible uncertainty. Therefore, the ranking conclusions need to be interpreted with caution. In addition, although this NMA suggests that PM combined with other TCM therapies may have better efficacy than simple PM, there is no evidence that more added therapies necessarily lead to better efficacy. This study only involved PM combined with one other TCM therapy, and the efficacy and safety of PM combined with multiple TCM therapies still need further study. The results of this study notwithstanding, blindly increasing the number of therapies is not recommended. In addition, what remains to be explored is the efficacy mechanism of PM combined with other TCM therapies and how to make the combination work best.

It is also worth noting that the GRADE assessment showed that outcomes such as overall response rate and total YGTSS score were rated as low or very low quality due to the limitations of the original study. Although our results, which are based on existing included studies, provide a degree of robustness, they need to be interpreted with caution. Our analysis suggested that PM + CHM may perform best in improving clinical efficacy, while PM + MA may perform best in reducing the total YGTSS score, but these conclusions still need further validation. Future RCTs should focus on methodological improvements, such as strengthening the blinding and randomization design, and improving the quality of reporting, so as to provide more solid and reliable evidence for the treatment of tic disorders in children.

### 4.3 Strengths and limitations

Several advantages of this study deserve to be emphasized. Most importantly, to the best of our knowledge, this is the first NMA to evaluate PM and its combination with other TCM therapies in the treatment of TD in children. Although previous systematic reviews have confirmed the safety and efficacy of PM in the treatment of TD, only a pairwise comparison between simple PM and WM or CHM has ever been performed (56, 62). In the clinical treatment of TD, PM is often not the only treatment means. This study assessed the efficacy and safety of PM and PM combined with other commonly used TCM therapies such as CHM, MA, AA, QT, CT, ST, and Mox, providing a more comprehensive, evidence-based foundation for selecting TCM treatment regimens for TD.

However, some limitations should be considered when interpreting our results. (1) First, despitestrict adherence to our inclusion and exclusion criteria and rigorous application of the Cochrane risk-of-bias tool, most eligible RCTs were single-center, small samples and demonstrated low methodological quality (e.g., unclear or high risk of bias in randomization, allocation concealment and blinding), which was attributed to inherent limitations in the available literature. The results of some outcomes changed after the exclusion of studies with high risk of bias, suggesting that the quality of the included studies may affect the robustness of the results. This should be considered when interpreting our findings. Therefore, future randomized controlled trials should pay attention to improving methodological rigor, such as implementing multicenter designs and standardizing randomization schedules and allocation concealment. (2) Second, there was heterogeneity in PM protocols among the included studies, including differences in core acupoints, manipulation, pressure, frequency, duration, and course of treatment. Subgroup analysis showed that the degree of standardization of PM operations may affect the results of the study. Additionally, there were differences in the diagnostic criteria used across the included studies. Although this situation reflects the characteristics of TCM syndrome differentiation and individualized treatment, the lack of standardized protocols may increase inconsistency. Future RCTs should adopt standardized consensus-based PM protocols and diagnostic criteria to improve the comparability and stability of the evidence. (3) Third, most of the included studies lacked long-term follow-up. Only two included studies (41, 45) included a half-year follow-up, and only one study (30) reported TD recurrence rates. Due to the lack of follow-up time, the persistence and recurrence risk of PM and related TCM therapy cannot be fully evaluated, which may affect the judgment of the long-term value of the therapy in clinical decision-making. Future studies need to extend the follow-up period and systematically collect recurrence data to comprehensively evaluate the long-term efficacy and safety. (4) In addition, it should be noted that all included RCTs were conducted in China, which may cause regional bias. Regional differences in patient demographics as well as attitudes toward PM therapy may limit the generalizability of our findings to other populations. (5) Finally, only one study (44) assessed patients' adherence by number of sessions, so our NMA was unable to quantify this key factor. Since adherence is particularly important in pediatric populations, future studies should infuse an assessment of adherence. Therefore, disease course, etiology, symptoms, and other factors should be fully considered in treating TD, and individual TCM therapies should be formulated on a case-by-case basis. In the future, high-quality, multi-center, large-sample RCTs should be conducted to provide more reliable evidence.

## 5 Conclusion

Our study indicates that PM and PM combined with other TCM therapies are effective and safe interventions for TD in children. PM + CHM may be the preferred intervention to improve the total effective rate and reduce YGTSS motor tics score and YGTSS vocal tics score for TD in children. PM + MA performs pretty well in reducing YGTSS total score. In addition, simple PM has significant advantages in reducing adverse reaction rates. However, there are still limitations to this study, such as the unclear or high risk of bias in the randomization and blinding of many included randomized controlled trials, which may affect the robustness of these findings. These conclusions thus need to be interpreted with caution and validated by high-quality, multi-center, large-sample RCTs.

## Data Availability

The original contributions presented in the study are included in the article/Supplementary Material, further inquiries can be directed to the corresponding authors.
