# Supplementary material for: Pediatric massage in conjunction with other traditional Chinese medicine therapies for tic disorder in children: systematic review and network meta-analysis
Source: Front Pediatr. 2025 Aug 26;13:1609934. doi: 10.3389/fped.2025.1609934 (PMC12417398; doi:10.3389/fped.2025.1609934)
Supplement: Supplementary file 2 [file Datasheet1.docx]

**Supplementary Material**

**Supplementary material 1: Results after excluding studies with high risk of bias**


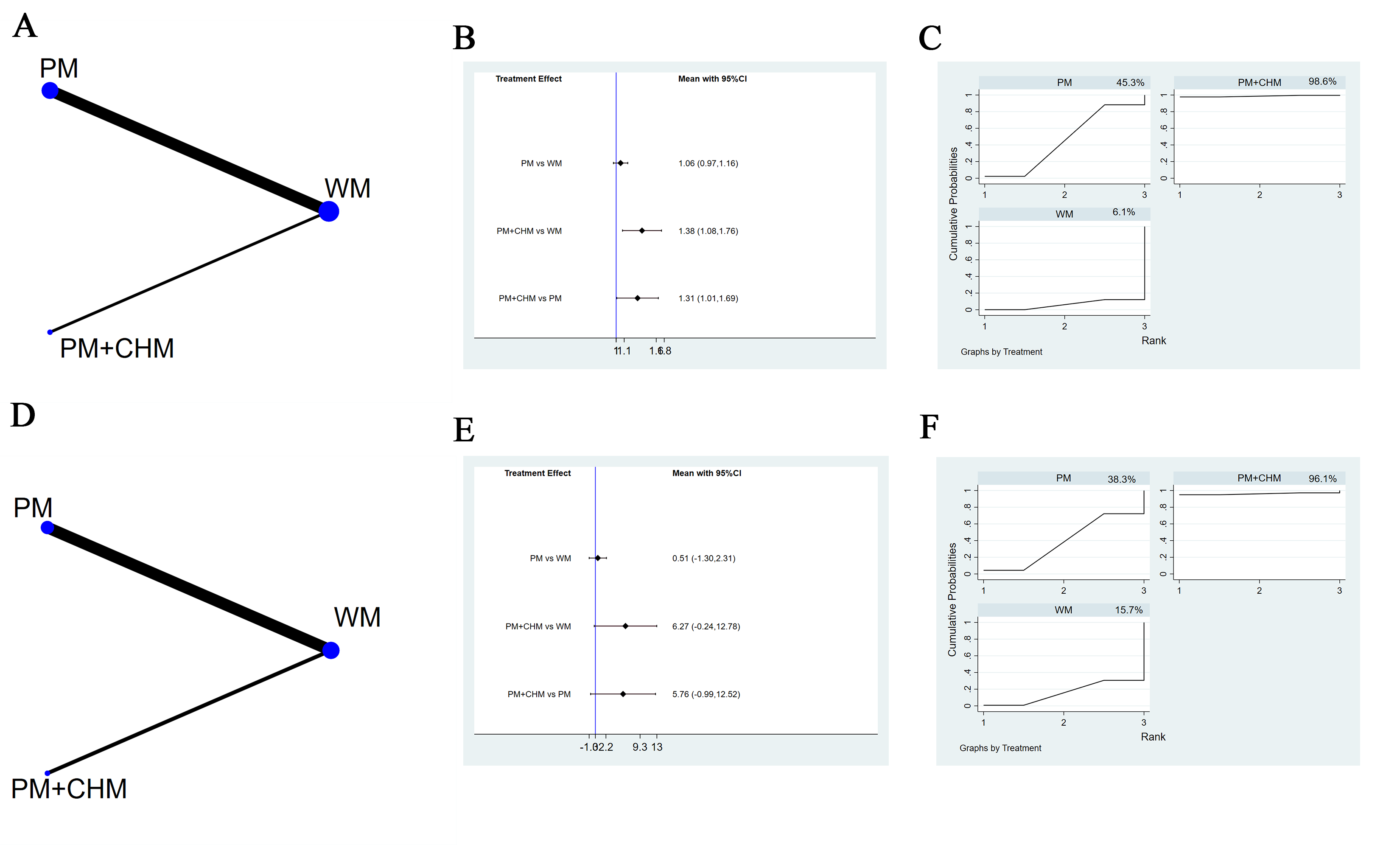


**Notes:** Network plots (A), forest map (B), and SUCRA values (C) for the total effective rate after excluding studies with high risk of bias. Network plots (D), forest map (E), and SUCRA values (F) for the total YGTSS score after excluding studies with high risk of bias.

**Abbreviations:** WM, Western medicine; PM, Pediatric massage; CHM, Chinese herbal medicine.

**Supplementary material 2: Results of subgroup analysis for the total effective rate according to the standardization degree of PM operations**


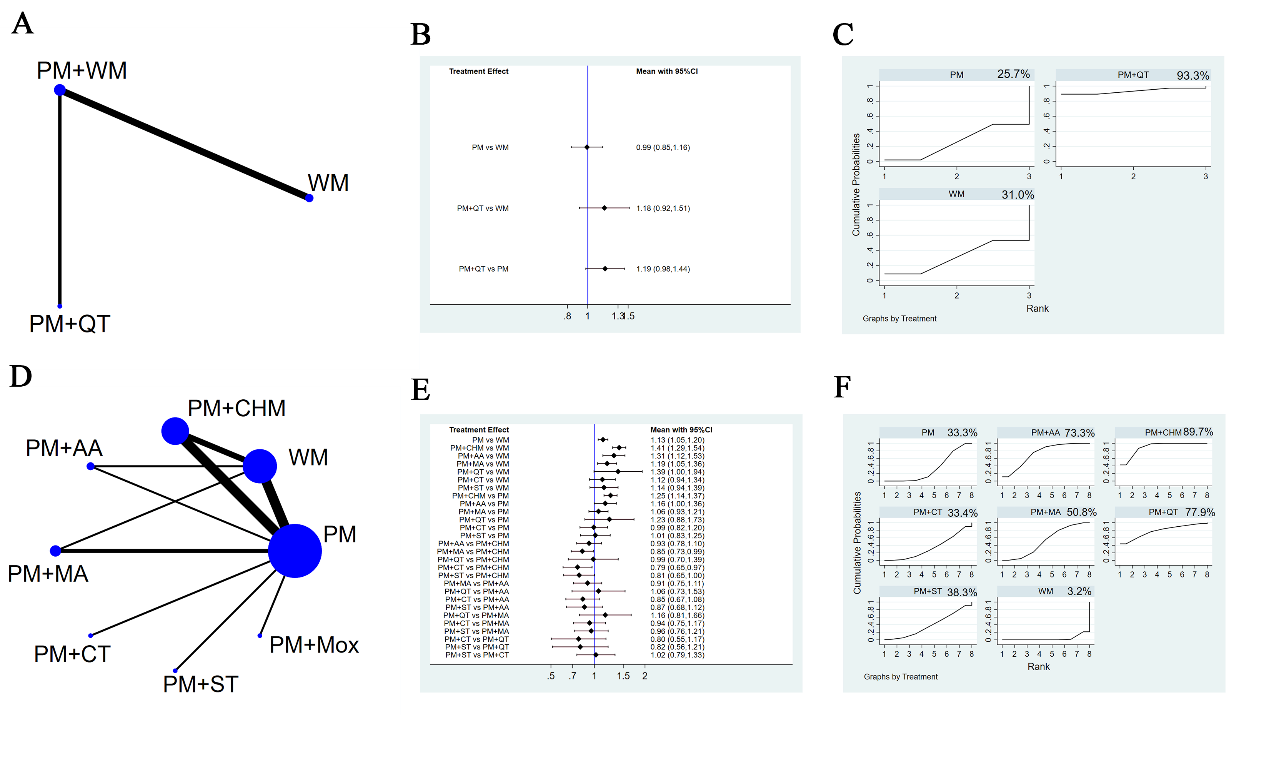


**Notes:** Network plots (A), forest map (B), and SUCRA values (C) for the total effective rate in the subgroup with a higher standardization degree of PM operations. Network plots (D), forest map (E), and SUCRA values (F) for the total effective rate in the subgroup with a lower standardization degree of PM operations.

**Abbreviations:** WM, Western medicine; PM, Pediatric massage; CHM, Chinese herbal medicine; MA, manual acupuncture; AA, auricular acupuncture; QT, Qigong therapy; CT, cupping therapy; ST, scraping therapy; Mox, moxibustion.

**Supplementary material 3: Results of subgroup analysis for the total effective rate according to the standardization degree of PM operations**


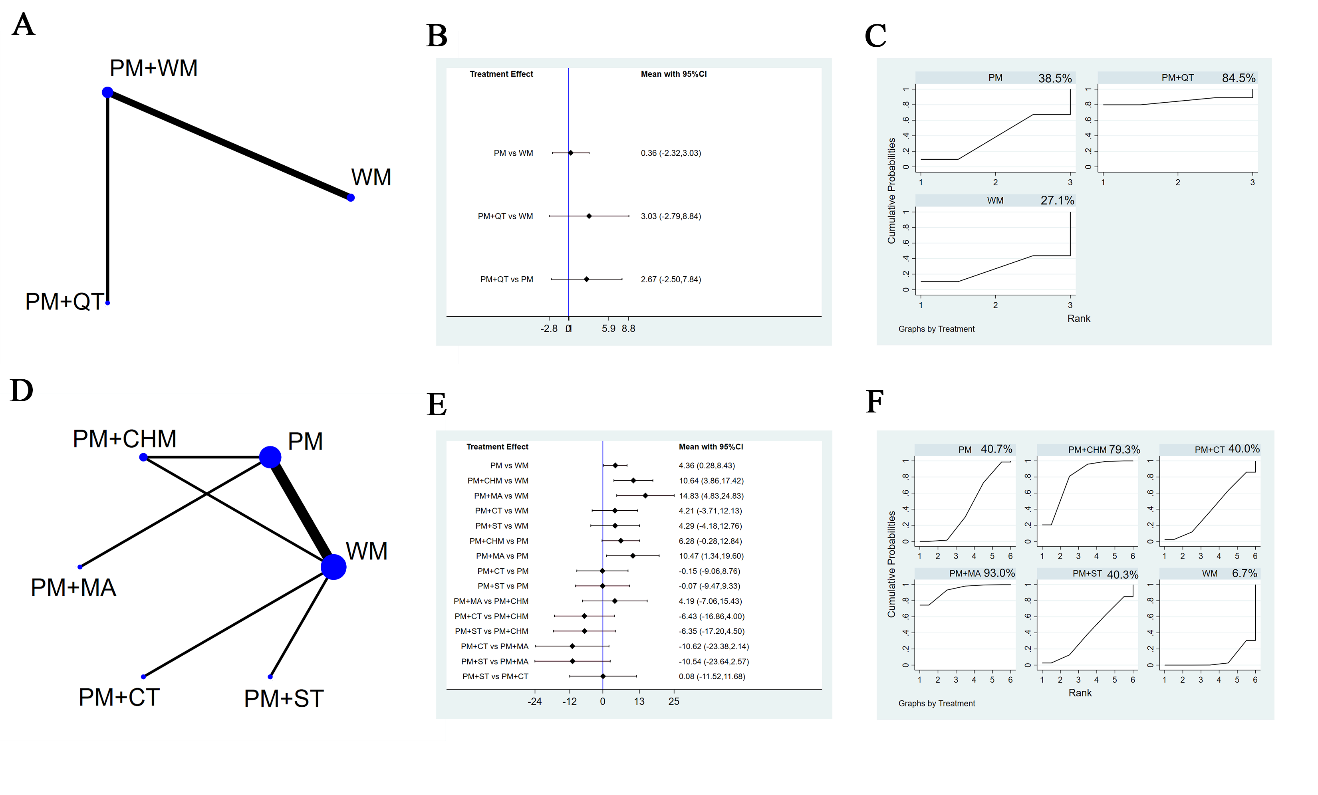


**Notes:** Network plots (A), forest map (B), and SUCRA values (C) for the total YGTSS score in the subgroup with a higher standardization degree of PM operations. Network plots (D), forest map (E), and SUCRA values (F) for the total YGTSS score in the subgroup with a lower standardization degree of PM operations.

**Abbreviations:** WM, Western medicine; PM, Pediatric massage; CHM, Chinese herbal medicine; MA, manual acupuncture; AA, auricular acupuncture; QT, Qigong therapy; CT, cupping therapy; ST, scraping therapy; Mox, moxibustion.
